# Electrostatic wave interaction via asymmetric vector solitons as precursor to rogue wave formation in non-Maxwellian plasmas

**DOI:** 10.1038/s41598-024-52431-7

**Published:** 2024-01-25

**Authors:** N. Lazarides, Giorgos P. Veldes, D. J. Frantzeskakis, Ioannis Kourakis

**Affiliations:** 1 Department of Mathematics, Khalifa University of Science and Technology, P.O. Box 127788, Abu Dhabi, United Arab Emirates; 2 Department of Physics, University of Thessaly, 35100 Lamia, Greece; 3 Department of Physics, National and Kapodistrian University of Athens, Zografou, 15784 Athens, Greece; 4 Space & Planetary Science Center, Khalifa University of Science and Technology, P. O. Box 127788, Abu Dhabi, United Arab Emirates; 5 Hellenic Space Center, Leoforos Kifissias 178, Chalandri, 15231 Athens, Greece

**Keywords:** Mathematics and computing, Physics

## Abstract

An asymmetric pair of coupled nonlinear Schrödinger (CNLS) equations has been derived through a multiscale perturbation method applied to a plasma fluid model, in which two wavepackets of distinct (carrier) wavenumbers ($k_1$ and $k_2$) and amplitudes ($\Psi _1$ and $\Psi _2$) are allowed to co-propagate and interact. The original fluid model was set up for a non-magnetized plasma consisting of cold inertial ions evolving against a $\kappa$-distributed electron background in one dimension. The reduction procedure resulting in the CNLS equations has provided analytical expressions for the dispersion, self-modulation and cross-coupling coefficients in terms of the two carrier wavenumbers. These coefficients present no symmetry whatsoever, in the general case (of different wavenumbers). The possibility for coupled envelope (vector soliton) solutions to occur has been investigated. Although the CNLS equations are asymmetric and non-integrable, in principle, the system admits various types of vector soliton solutions, physically representing nonlinear, localized electrostatic plasma modes, whose areas of existence is calculated on the wavenumbers’ parameter plane. The possibility for either bright (B) or dark (D) type excitations for either of the (2) waves provides four (4) combinations for the envelope pair (BB, BD, DB, DD), if a set of explicit criteria is satisfied. Moreover, the soliton parameters (maximum amplitude, width) are also calculated for each type of vector soliton solution, in its respective area of existence. The dependence of the vector soliton characteristics on the (two) carrier wavenumbers and on the spectral index $\kappa$ characterizing the electron distribution has been explored. In certain cases, the (envelope) amplitude of one component may exceed its counterpart (second amplitude) by a factor 2.5 or higher, indicating that extremely asymmetric waves may be formed due to modulational interactions among copropagating wavepackets. As $\kappa$ decreases from large values, modulational instability occurs in larger areas of the parameter plane(s) and with higher growth rates. The distribution of different types of vector solitons on the parameter plane(s) also varies significantly with decreasing $\kappa$, and in fact dramatically for $\kappa$ between 3 and 2. Deviation from the Maxwell-Boltzmann picture therefore seems to favor modulational instability as a precursor to the formation of bright (predominantly) type envelope excitations and freak waves.

## Introduction

A pair of nonlinearly coupled nonlinear Schrödinger equations (hereafter referred to as the CNLS system of equations) arises as a prototype model of mathematical physics, which occurs in various physical contexts^1–4^, including water waves^5,6^, left-handed (negative refraction index) transmission lines^7^, optical pulse propagation in birefringence fibers^8–10^, and in optical nonlinear media^11^, vector solitons in left-handed metamaterials^12^, polarized pulse pair propagation in anisotropic dispersive media^13^, in electrically driven graphene multilayer mediums^14^, pulse propagation in isotropic Kerr media with chromatic dispersion^15^, and even breathers and rogue waves in optical fibers^16^. Formally similar systems of equations have been used to model light (beam) propagation^17–20^ and electrostatic or electromagnetic wave propagation in plasmas^21–28^. Independently from a physical context, various studies of vector solitons and rogue waves have been carried out, based on general CNLS models^6,29–34^, and variants of CNLS equations such as coupled derivative nonlinear Schrödinger equations^35,36^, vector ($N$-component) CNLS^37–39^, nonlocal CNLS^40^, CNLS equations with variable coefficients^41^, coherently coupled CNLS equations^42^, and coupled high-order nonlinear Schrödinger equations^43^, among others, have been investigated with respect to vector solitons and rogue waves.

A pair of nonlinearly coupled CNLS equations was recently^27,28^ derived from a plasma model consisting of a cold inertial ion fluid evolving against an electron background. Generalizing an earlier study involving a thermal (Maxwellian) electron distribution^27^, the formalism has been recently^28^ adopted to electron population(s) that follows a kappa distribution^44–51^. The kappa (family of) distribution functions (DF) is characterized by a spectral index $\kappa$, and exhibits a high-energy tail in the large (suprathermal) range of electron velocities. The analytical expression of the kappa DF converges to the Maxwell-Boltzmann distribution for infinite $\kappa$. Such distributions are a common occurrence in Space plasma observations, e.g. in the solar wind^44,45,52^ and in planetary magnetospheres^53^. The original plasma fluid model and its lengthy algebraic reduction to a pair of CNLS equations for the envelopes of two modulated electrostatic wavepackets, by using a Newell type multi-scale perturbation technique, is described in great detail in Refs.^27,28^, so the details will be omitted in the following. The main outcome of that study, in the form of the (six) coefficients involved in the resulting CNLS equations, will be presented here, for completeness, in terms of the wavenumbers of the two interacting waves and the spectral index $\kappa$ which characterizes the electron distribution. Based on the system of CNLS equations obtained in those earlier studies, our ambition in the paper at hand is to investigate the existence of coupled localized envelope modes (vector solitons), from first principles, and to explore their dependence on the intrinsic plasma parameters, namely the two wavenumbers ($k_1$, $k_2$) and $\kappa$.

It must be pointed out that the CNLS system of equations that forms the basis of our study is *not* amenable to the widely studied (integrable) Manakov system^54^, unless identical carrier waves ($k_1=k_2$) are considered. Indeed, for arbitrary wavenumbers of the two co-propagating waves, the coefficients of the CNLS equations do not exhibit any known symmetry, hence the system is rendered non-symmetric. For the same reason, any attempt for reduction of the number of the coefficients of these CNLS equations cannot give less than four coefficients^55,56^.

The CNLS system of equations in its general form admits vector soliton solutions, rogue waves, and breathers, which can be obtained either analytically or numerically. In this paper, within the context of the plasma fluid model considered for electrostatic waves, we have obtained four different types of vector solitons, whose components are actually combinations of bright and dark type envelope solitons, i.e. reminiscent of solutions of the single nonlinear Schrödinger equation. We have derived a set of analytical conditions for the existence of such vector solitons, in terms of the various coefficients (assumed to take arbitrary values), and we have subsequently explored their parametric dependence on the relevant plasma parameters: the two wavevectors and the spectral index $\kappa$. In each of these existence regions, on the ($k_1,k_2$) plane, we have also calculated the vector soliton parameters, i.e., the envelope amplitudes and their (common) width. These (amplitude and width) obviously vary, upon a variation of either of the wavenumbers or the spectral index $\kappa$. Several illustrative examples are shown in the following, in which a structural transition between a particular type of a vector soliton (and its parameters) and another can be observed. These transition may either be smooth, or take place through a divergence of the width and the amplitudes at a transition point (boundary between different regions). In certain cases, one of the components of a vector soliton may acquire a very high amplitude with respect to that of its sister component. We may characterize these solutions as extremely asymmetric vector solitons, a configuration which has not been discussed before.

## Asymmetric nonlinear Schrödinger equations and coefficients

A plasma fluid model was considered in earlier work^28^, as a basis to describe electrostatic (ion-acoustic) waves. A non-magnetized plasma was considered, consisting of a cold inertial ion fluid evolving against a “thermalized” (highly energetic) electron background, in a one-dimensional geometry. Given the large mass disparity between the electrons and the massive ions, the former were assumed to be inertia-less, thus characterized by an equilibrium configuration, modeled as a $\kappa$-type velocity distribution. Two co-propagating wavepackets were considered, with wavevectors $k_1$ and $k_2$, respectively. A Newell type multiple scale perturbation technique led, after a tedious calculation^28^, to the following pair of CNLS equations:

1 $$\begin{aligned}{} & {} i \left( \frac{\partial \Psi _1}{\partial t_2} +v_{g,1} \frac{\partial \Psi _1}{\partial x_2} \right) +P_1 \frac{\partial ^2 \Psi _1}{\partial x_1^2} +\left( Q_{11} |\Psi _1|^2 +Q_{12} |\Psi _2|^2 \right) \Psi _1 =0, \end{aligned}$$

2 $$\begin{aligned}{} & {} i \left( \frac{\partial \Psi _2}{\partial t_2} +v_{g,2} \frac{\partial \Psi _2}{\partial x_2} \right) +P_2 \frac{\partial ^2 \Psi _2}{\partial x_1^2} +\left( Q_{21} |\Psi _1|^2 +Q_{22} |\Psi _2|^2 \right) \Psi _2 =0 \,, \end{aligned}$$

where

3 $$\begin{aligned} P_j =-\frac{3}{2} \frac{c_1 k_j}{(k_j^2 +c_1)^{5/2}} = \frac{1}{2} \frac{\partial ^2 \omega _j}{\partial k_j^2}, \qquad v_{g,j} =\frac{c_1}{(k_j^2 +c_1)^{3/2}} =\frac{\partial \omega _j}{\partial k_j}, \qquad \omega _j =\frac{k_j}{\sqrt{k_j^2 +c_1}} \end{aligned}$$

are the (linear) dispersion coefficients, the group velocities, and the frequency dispersion relations, respectively. Note that $P_j = \frac{1}{2}\frac{\partial ^2 \omega _j}{\partial k_j^2}$, in a way formally analogous to the group-velocity-dispersion (GVD) terms known in nonlinear optics. $Q_{11}$ and $Q_{22}$ are self-modulation coefficients and $Q_{12}$ and $Q_{21}$ are cross-coupling coefficients. For the exact expressions providing the (four) coefficients $Q_{ij}$ (for $i, j =$ 1 or 2) as functions of $k_1$, $k_2$ and $\kappa$ (via the constants $c_1$, $c_2$, and $c_3$), see the Supplementary Information part accompanying this article. We emphasize that, unless $k_1 = k_2$ (a stringent condition that won’t be satisfied, in general), the above pairs of coefficients take different values, viz. $P_1 \ne P_2$, $Q_{11} \ne Q_{22}$ and $Q_{12} \ne Q_{21}$.

The constants $c_1$, $c_2$, and $c_3$, incorporating the effect of $\kappa$, are given as by^57^

4 $$\begin{aligned} c_1 =\frac{\kappa -\frac{1}{2}}{\kappa -\frac{3}{2}}, \qquad c_2 =\frac{\left( \kappa -\frac{1}{2}\right) \left( \kappa +\frac{1}{2}\right) }{2! \left( \kappa -\frac{3}{2} \right) ^2}, \qquad c_3 =\frac{\left( \kappa -\frac{1}{2}\right) \left( \kappa +\frac{1}{2}\right) \left( \kappa +\frac{3}{2}\right) }{3! \left( \kappa -\frac{3}{2}\right) ^3}. \end{aligned}$$

The above expressions are the first three coefficients in the Mc Laurin expansion $n_e \simeq 1 +c_1 \phi +c_2 \phi ^2 +c_3 \phi ^3 + \cdots$, of the electron number density

5 $$\begin{aligned} n_e =\left( 1 -\frac{\phi }{\kappa -\frac{3}{2}} \right) ^{-\left( \kappa -\frac{1}{2} \right) }, \end{aligned}$$

that is obtained upon integrating the kappa distribution^44,52,58^, actually a straightforward replacement for the Maxwell-Boltzmann distribution when dealing space and astrophysical plasmas^47,59^. The nature and composition of the plasmas allows for extracting the spectral index $\kappa$ in each particular case. Indeed, kappa distributions with $2< \kappa < 6$ have been found to fit the observations and satellite data in the solar wind^60^, among other successful examples of data fitting in space plasmas, making the kappa distribution an ubiquitous paradigm in Space science^52^.

The CNLS equations (1) and (2) can be transformed to

6 $$\begin{aligned} i \left( \frac{\partial \Psi _1}{\partial \tau } +\delta \frac{\partial \Psi _1}{\partial \xi } \right) +P_1 \frac{\partial ^2 \Psi _1}{\partial \xi ^2} +\left( Q_{11} |\Psi _1|^2 +Q_{12} |\Psi _2|^2 \right) \Psi _1 =0, \end{aligned}$$

7 $$\begin{aligned} i \left( \frac{\partial \Psi _2}{\partial \tau } -\delta \frac{\partial \Psi _2}{\partial \xi } \right) +P_2 \frac{\partial ^2 \Psi _2}{\partial \xi ^2} +\left( Q_{21} |\Psi _1|^2 +Q_{22} |\Psi _2|^2 \right) \Psi _2 =0, \end{aligned}$$

after a change of the independent variables *x* and *t* (in which the subscripts have been dropped) through $\xi = x -v t$ and $\tau =t$, with $v =( v_{g,1} +v_{g,2} )/2$ and $\delta =( v_{g,1} -v_{g,2} )/2$ being is the half-sum and the half-difference of the group velocities, respectively.

Note that the “walk-off” parameter $\delta$ is, by its definition, clearly a function of both $k_1$ and $k_2$ (in addition to $\kappa$), through the group velocities $v_{g,j}$ ($j=1,2$) of the two interacting wavepackets in the plasma. Generally speaking, a large $\delta$ would be able to cause dynamic instabilities in the system and eventually prevent the formation of various types of vector solitons (to be discussed later in this article). However, from the expression of the group velocities $v_{g,j}$ in Eq. (3), we may observe that their values are limited in the interval $[- 1/\sqrt{c_1}, + 1/\sqrt{c_1}]$, where $c_1 =c_1(\kappa )$—defined in (4) above—exceeds unity. It follow that, in the Maxwellian case ($\kappa =100$), $c_1 =1$ and the extremal values of $\delta$ are $\pm 0.5$ (assuming co-propagating wavepackets), while for lower values of $\kappa$, the corresponding extremal values of $\delta$ are (in absolute value) even smaller, all the way down to zero (attained for $\kappa = 3/2$). Therefore, the walk-off parameter acquires small values, and is not expected to prevent soliton formation. Then, by applying the transformation $\Psi _1 = \bar{\Psi }_1\, \exp \left[ i \left( \frac{\delta ^2}{4 P_1} \tau -\frac{\delta }{2 P_1} \xi \right) \right]$ and $\Psi _2 = \bar{\Psi }_2\, \exp \left[ i \left( \frac{\delta ^2}{4 P_2} \tau +\frac{\delta }{2 P_2} \xi \right) \right]$ to Eqs. (6) and (7) we obtain the more familiar form

8 $$\begin{aligned} i \frac{\partial \bar{\Psi }_1}{\partial \tau } +P_1 \frac{\partial ^2 \bar{\Psi }_1}{\partial \xi ^2} +\left( Q_{11} |\bar{\Psi }_1|^2 +Q_{12} |\bar{\Psi }_2|^2 \right) \bar{\Psi }_1 =0, \end{aligned}$$

9 $$\begin{aligned} i \frac{\partial \bar{\Psi }_2}{\partial \tau } +P_2 \frac{\partial ^2 \bar{\Psi }_2}{\partial \xi ^2} +\left( Q_{21} |\bar{\Psi }_1|^2 +Q_{22} |\bar{\Psi }_2|^2 \right) \bar{\Psi }_2 =0, \end{aligned}$$

where $\bar{\Psi }_j$ are complex functions of the new variables $\xi$ and $\tau$.

## Modulational instability: compatibility condition and growth rate

Modulational instability (MI) analysis for two co-propagating plane-wave solutions of the CNLS equations (6) and (7) can be performed using the procedure described e.g., in Refs.^61^. The plane waves

10 $$\begin{aligned} \Psi _j =\Psi _{j,0} \, e^{i \tilde{\omega }_j \tau }, \end{aligned}$$

where $\Psi _{j,0}$ ($j=1,2$) is a constant real amplitude and $\tilde{\omega }_j$ is an internal frequency, are solutions of the CNLS equations for $\tilde{\omega }_1 =Q_{11} \Psi _{1,0}^2 +Q_{12} \Psi _{2,0}^2$ and $\tilde{\omega }_2 =Q_{21} \Psi _{1,0}^2 +Q_{22} \Psi _{2,0}^2$, and constitute nonlinear modes that may become modulationally unstable in the presence of a small amplitude perturbation of wavenumber *K* and perturbation frequency $\Omega$. By following the standard approach, we find that the above nonlinear modes are unstable whenever the *compatibility condition*

11 $$\begin{aligned} \left[ (\Omega -\delta K)^2 -\Omega _1^2 \right] \left[ (\Omega +\delta K)^2 -\Omega _2^2 \right] =\Omega _c^4, \end{aligned}$$

where $\Omega _j^2 =P_j K^2 \left( P_j K^2 -2 Q_{jj} \Psi _{j,0}^2 \right)$ and $\Omega _c^4 =4 P_1 P_2 Q_{12} Q_{21} \Psi _{1,0}^2 \Psi _{2,0}^2 K^4$, has at least one pair of complex conjugate roots ($j=1,2$). Then, the (positive) imaginary part of these roots provides the *growth rate* $\Gamma$ of the modulationally unstable modes. When Eq. (11) has two pairs of complex conjugate roots, then the largest of their imaginary parts provide the growth rate of the modulationally unstable modes, i.e.,

12 $$\begin{aligned} \Gamma =\textrm{max}\{\textrm{Im}(\Omega _r)\}, \end{aligned}$$

where $\Omega _r$ denotes one of the four roots of the compatibility condition above.

The growth rate $\Gamma$ is calculated numerically for the CNLS equations by finding the roots of the fourth degree (in $\Omega$) polynomial resulting from the compatibility condition, Eq. (11). Then $\Gamma$ is mapped on the $k_1 - k_2$ parameter plane for several values of the perturbation wavenumber *K*, and two values of the spectral index $\kappa$, i.e., for $\kappa =2$ and $\kappa =3$. The amplitudes of the nonlinear wave modes in these calculations are $\Psi _{1,0} =\Psi _{2,0} =0.1$.

**Figure 1:**
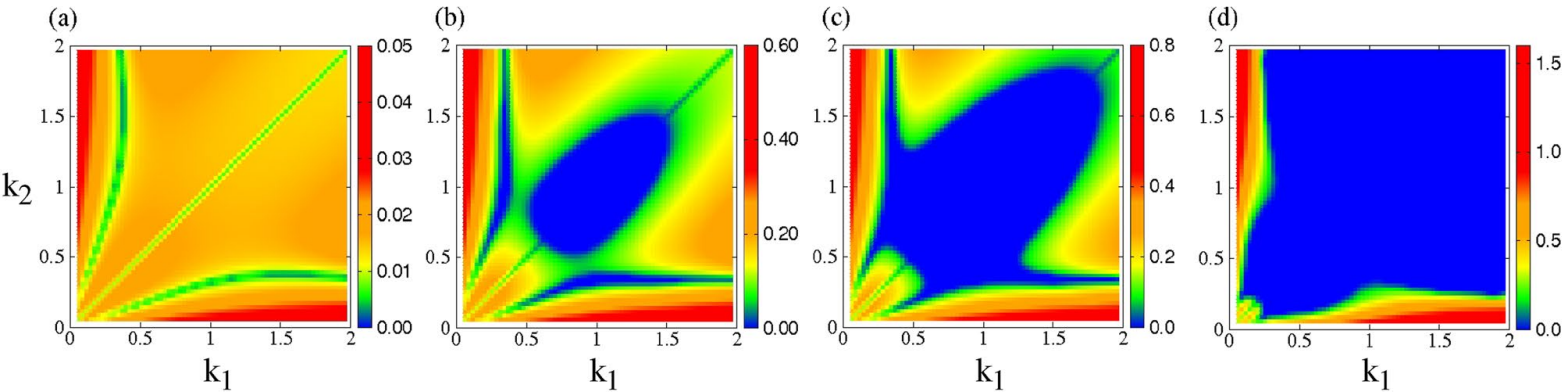
Maps of the growth rate $\Gamma$ on the $k_1 - k_2$ plane for $\kappa =2$, $\Psi _{1,0} =\Psi _{2,0} =0.1$, and perturbation wavenumber (**a**) $K =0.1$; (**b**) $K =1.3$; (**c**) $K =1.6$; (**d**) $K =3.4$. The values of *K* are chosen so that they illustrate the variability of the growth rate patterns in the best possible way.

**Figure 2:**
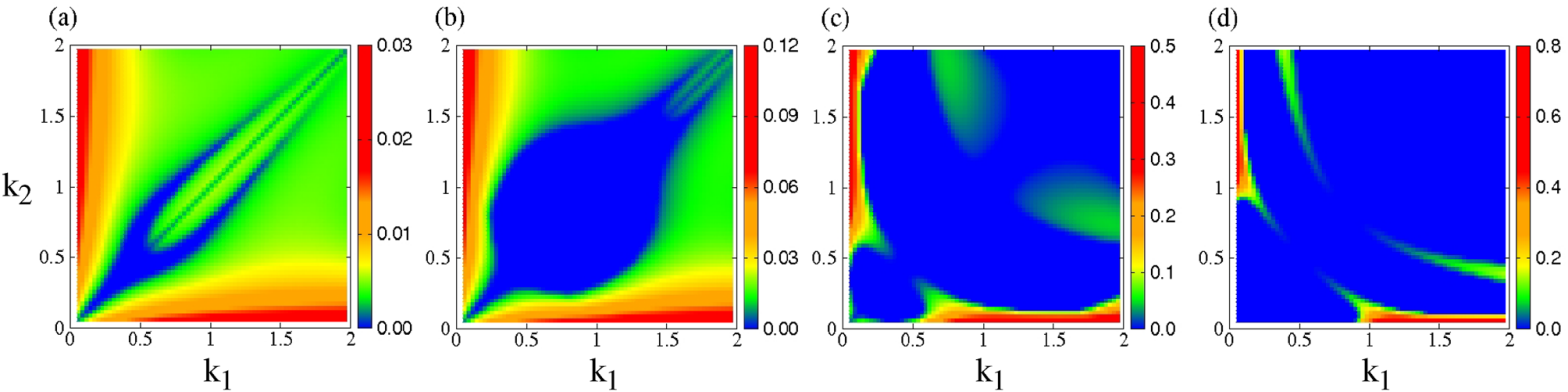
Maps of the growth rate $\Gamma$ on the $k_1 - k_2$ plane for $\kappa =3$, $\Psi _{1,0} =\Psi _{2,0} =0.1$, and perturbation wavenumber (**a**) $K =0.1$; (**b**) $K =0.4$; (**c**) $K =1.9$; (**d**) $K =3.4$. The values of *K* are chosen so that they illustrate the variability of the growth rate patterns in the best possible way.

In Figs. 1 and 2, maps of the growth rate $\Gamma$ are shown on the $k_1 - k_2$ parameter plane of the wavenumbers of the two co-propagating (carrier) waves for spectral index $\kappa =2$ and $\kappa =3$, respectively. These values of $\kappa$ were selected to be into the range of physically acceptable values for space plasmas that have been observed to be in the interval from $\kappa =2$ to $\kappa =6$. Moreover, they are more or less symmetrically arranged around the value $\kappa \simeq 2.5$. In Fig. 1, in particular, for $\kappa =2$, the system is modulationally unstable for low values of the perturbation wavenumber *K*, for all the parameter plane shown (Fig. 1a). At around $K \approx 1.2$, the first modulationally stable island becomes visible, which acquires substantial area roughly in the middle of the $k_1 - k_2$ parameter plane for $K=1.3$ (blue color, Fig. 1c). Note that there are also two narrow modulationally stable (blue) areas develop around the curves where $Q_{12}$ and $Q_{21}$ are zero. For *K* larger than 1.3, the modulationally stable areas grow and merge together into a single large one, which continues to grow with increasing *K* (Fig. 1c,d). For *K* larger than 3.4, almost all of the $k_1 - k_2$ parameter plane shown in Fig. 1 becomes modulationally stable. Note the strong modulational instability in Fig. 1d that is limited at low $k_1$ and low $k_2$, where the nonlinear coupling coefficients $Q_{12}$ and $Q_{21}$ acquire very large values.

In Fig. 2, for $\kappa =3$, the system possess a modulationally stable area even for low values of *K*, as e.g., can be observed in Fig. 2a (blue color), which is close and around the diagonal $k_1 =k_2$. Already at $K =0.4$, the modulationally unstable area has grown significantly (Fig. 2b), while it continues to grow with increasing value of *K* (Fig. 2c,d). For *K* larger than 3.4 almost all of the parameter plane shown is modulationally stable. Again, the strongly unstable areas are limited in the low $k_1$ and low $k_2$ areas of the plane.

## Vector solitons: existence and their parameters

The CNLS equations Eqs. (8) and (9) admit several types of vector soliton solutions that are combinations of bright and dark soliton solutions of the single NLS equation. Indeed, as we shall show below, four types of vector solitons, i.e., bright-bright (BB), bright-dark (BD), dark-bright (DB), ans dark-dark (DD), may exist in the CNLS system Eqs. (8) and (9). Their parameters, i.e., their amplitudes $A_1$ and $A_2$, their (common) width *b*, and their internal frequencies $\nu _1$ and $\nu _2$ are related through simple mathematical expressions to the coefficients $P_j$ and $Q_{ij}$ of the CNLS equations (8) and (9). Each component of these vector solitons represent modulated electrostatic wavepackets which are moving in the plasma and interact strongly and nonlinearly with strengths $Q_{12}$ and $Q_{21}$.

The four types of vector solitons that may exist in the system of CNLS equations are given below along with their parameters, i.e., their amplitudes $A_1$ and $A_2$ and their width *b*, as a function of the CNLS coefficients $P_j$ and $Q_{ij}$ ($j=1,2$) which are in turn functions of the wavenumbers $k_1$ and $k_2$. Note that in the vector solitons expressions below one of the parameters may take arbitrary values. Here, we choose the amplitude $A_1$ as the free parameter and fix it to $A_1 =0.1$ in what follows.

*Case I: Bright-bright (BB) vector solitons.*

We seek for bright-bright (BB) vector solitons in the form

13 $$\begin{aligned} \bar{\Psi }_1 =A_1\, \textrm{sech}\left( b \xi \right) e^{-i \nu _1 \tau }, \qquad \bar{\Psi }_2 =A_2\, \textrm{sech}\left( b \xi \right) e^{-i \nu _2 \tau }. \end{aligned}$$

By substitution of Eq. (13) into Eqs. (8) and (9) we obtain

14 $$\begin{aligned} \left( {A_2}/{A_1} \right) ^2 =-\alpha , \qquad \left( {b}/{A_1} \right) ^2 =-\beta , \end{aligned}$$

and $$\nu _1 =-b^2 P_1$$, $$\nu _2=-b^2 P_2$$, where

15 $$\begin{aligned} \alpha =\frac{Q_{21} P_1 -Q_{11} P_2}{P_1 Q_{22} -P_2 Q_{12}}, \qquad \beta =\frac{1}{2} \frac{Q_{11} Q_{22} -Q_{21} Q_{12}}{P_2 Q_{12} -P_1 Q_{22}}. \end{aligned}$$

*Case II: Bright-dark (BD) vector solitons.*

We seek for bright-dark (BB) vector solitons in the form

16 $$\begin{aligned} \bar{\Psi }_1 =A_1\, \textrm{sech}\left( b \xi \right) e^{-i \nu _1 \tau }, \qquad \bar{\Psi }_2 =A_2\, \tanh \left( b \xi \right) e^{-i \nu _2 \tau }. \end{aligned}$$

By substitution of Eq. (16) into Eqs. (8) and (9) we obtain

17 $$\begin{aligned} \left( {A_2}/{A_1} \right) ^2 =+\alpha , \qquad \left( {b}/{A_1} \right) ^2 =-\beta , \end{aligned}$$

and $\nu _1 =-b^2 P_1 -Q_{12} A_2^2$, $\nu _2=-Q_{22} A_2^2$, where $\alpha$ and $\beta$ are given by Eq. (15).

*Case III: Dark-bright (DB) vector solitons.*

We seek for dark-bright (DB) vector solitons in the form

18 $$\begin{aligned} \bar{\Psi }_1 =A_1\, \tanh \left( b \xi \right) e^{-i \nu _1 \tau }, \qquad \bar{\Psi }_2 =A_2\, \textrm{sech}\left( b \xi \right) e^{-i \nu _2 \tau }. \end{aligned}$$

By substitution of Eq. (18) into Eqs. (8) and (9) we obtain

19 $$\begin{aligned} \left( {A_2}/{A_1} \right) ^2 =+\alpha , \qquad \left( {b}/{A_1} \right) ^2 =+\beta , \end{aligned}$$

and $\nu _1 = - \left( Q_{11} A_1^2 +Q_{12} A_2^2\right)$, $\nu _2= - \left( b^2 P_2 +Q_{21} A_1^2\right)$, where $\alpha$ and $\beta$ are given by Eq. (15).

*Case IV: Dark-dark (DD) vector solitons.*

We seek for dark-bright (DB) vector solitons in the form

20 $$\begin{aligned} \bar{\Psi }_1 =A_1\, \tanh \left( b \xi \right) e^{-i \nu _1 \tau }, \qquad \bar{\Psi }_2 =A_2\, \tanh \left( b \xi \right) e^{-i \nu _2 \tau }. \end{aligned}$$

By substitution of Eq. (20) into Eqs. (8) and (9) we obtain

21 $$\begin{aligned} \left( {A_2}/{A_1} \right) ^2 =-\alpha , \qquad \left( {b}/{A_1} \right) ^2 =+\beta , \end{aligned}$$

and $\nu _1 =- \left( Q_{11} A_1^2 +Q_{12} A_2^2 \right)$, $\nu _2 =-\left( Q_{21} A_1^2 +Q_{22} A_2^2 \right)$, where $\alpha$ and $\beta$ are given by Eq. (15).

Thus, the vector soliton parameters $A_j$, *b*, and $\nu _j$ are actually themselves functions of the wavenumbers $k_1$ and $k_2$ of the two co-propagating wavepackets through the CNLS coefficients $P_j$ and $Q_{ij}$ ($j=1,2$). The simplest way to calculate them is to first fix one of them (one of the amplitudes selected as reference, say $A_1$), and then calculate $A_2$ and *b* (e.g., from Eq. (21) in Case IV), and last the frequencies $\nu _j$.

For BB, BD, DB and DD vector solitons to exist in the CNLS system that we have obtain from the particular plasma fluid model considered here, the right-hand-sides of Eqs. (14), (17), (19), and (21), respectively, must be greater than zero (so that $A_2$ and *b* are real; $A_1$ is of course fixed to a real number). This condition for the existence of the four types of vector solitons can be expressed simply as

22 $$\begin{aligned}&\alpha< 0, \, \, \, \beta < 0, \, \, \,&\mathrm{Case~I, ~BB ~vector ~solitons,} \end{aligned}$$

23 $$\begin{aligned}&\alpha > 0, \, \, \, \beta < 0, \, \, \,&\mathrm{Case~II, ~BD ~vector ~solitons,} \end{aligned}$$

24 $$\begin{aligned}&\alpha> 0,\, \, \, \beta > 0, \, \, \,&\mathrm{Case~III, ~DB ~vector ~solitons,} \end{aligned}$$

25 $$\begin{aligned}&\alpha < 0, \, \, \, \beta > 0, \, \, \,&\mathrm{Case~IV, ~DD ~vector ~solitons \,.} \end{aligned}$$

**Figure 3:**
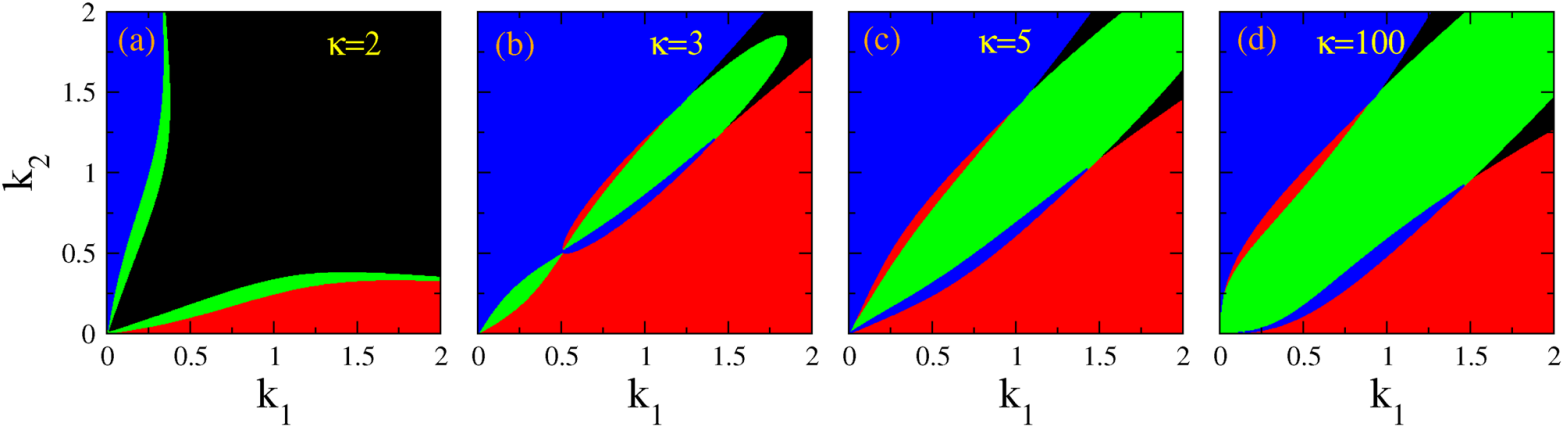
Existence areas for bright-bright (BB, black color), bright-dark (BD, red color), dark-bright (DB, blue color), and dark-dark (DD, green color), on the $k_1 - k_2$ parameter plane, for $A_1 =0.1$ and (**a**) $\kappa =2$; (**b**) $\kappa =3$; (**c**) $\kappa =5$; (**d**) $\kappa =100$.

**Figure 4:**
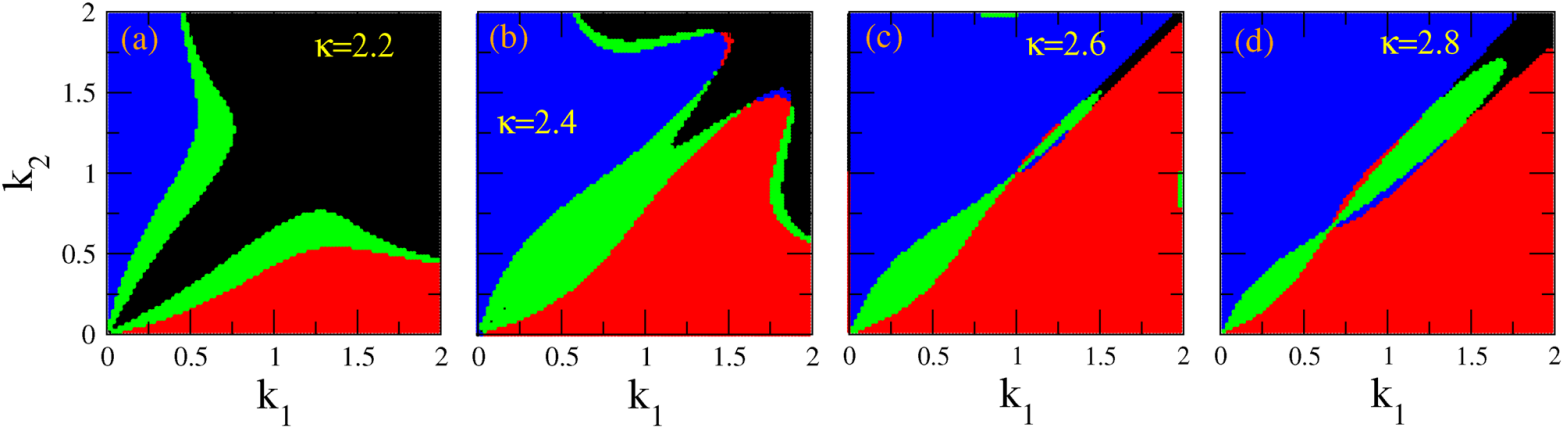
The same as Fig. 3 for values of $\kappa$ between 2 and 3. Existence areas for bright-bright (BB, black color), bright-dark (BD, red color), dark-bright (DB, blue color), and dark-dark (DD, green color), on the $k_1 - k_2$ space, for $A_1 =0.1$, and (**a**) $\kappa =2.2$; (**b**) $\kappa =2.4$; (**c**) $\kappa =2.6$; (**d**) $\kappa =2.8$.

In Fig. 3, we have identified on the $k_1 - k_2$ plane those areas in which each type of vector soliton may exist, for several values of the spectral index $\kappa$ spanning a very wide range from 2 to 100 (for the latter, the kappa distribution has practically converged to a Maxwell-Boltzmann one). In Fig. 3a, the black color that corresponds to BB vector solitons is dominant, and occupies a large part of the plane except that for low $k_1$ and $k_2$ where BD (red color) and DB (blue color) vector solitons may exist. Note that between the areas of existence of BB and DB, as well as between BB and BD, DD vector solitons exist (green color) in two separate narrow areas. Interestingly, the latter are grown around the path where $Q_{12}$ and $Q_{21}$ are zero on the $k_1 - k_2$ plane. For $\kappa =3$ or greater, different patterns of existence of vector solitons appear (Fig. 3b), which typically consist of two large areas of existence of BD and DB vector solitons (red and blue color, respectively) while in between of these two there is a green area in which DD vector solitons exist. A small black area in which BB solitons may exist also appears at the upper right corner of the plane. This pattern appears for all larger values of $\kappa$, with only slight quantitative differences observed in Fig. 3c,d. Obviously, the strongest variation of the existence patterns of vector solitons on the $k_1 - k_2$ plane occurs between $\kappa =2$ and $\kappa =3$. In order to analyze this pattern variability, we present in Fig. 4 a series of patterns for four (4) values of $\kappa$ between 2 and 3. Here we see how the dominant (black) area of BB vector solitons gradually shrinks against both the existence areas for BD and DB vector solitons (red and blue area, respectively).

**Figure 5:**
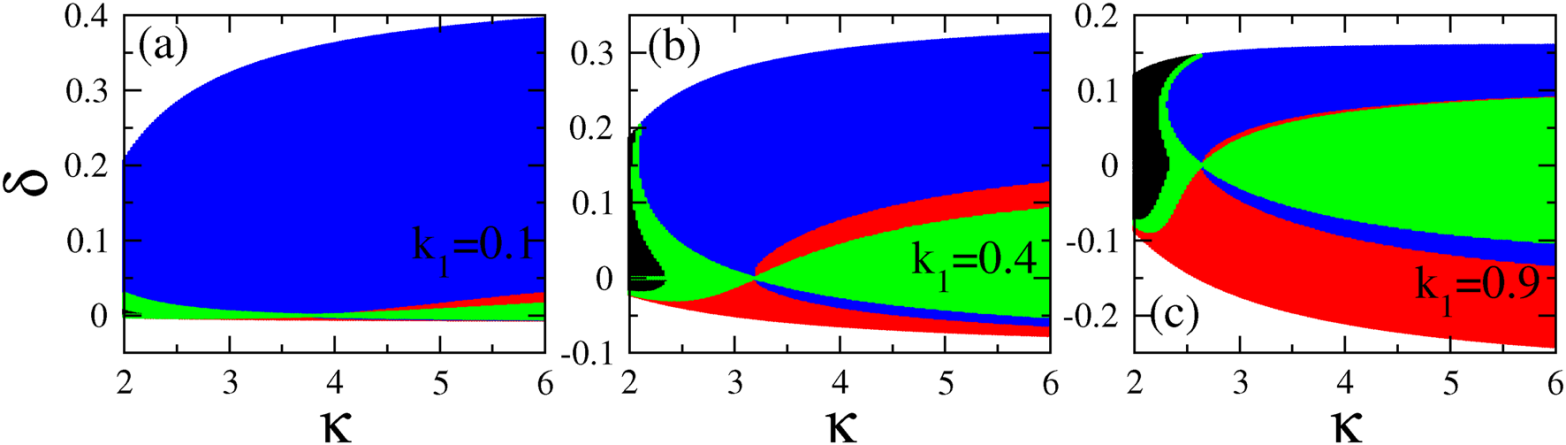
Existence areas for bright-bright (BB, black color), bright-dark (BD, red color), dark-bright (DB, blue color), and dark-dark (DD, green color), as a function of $\kappa$ and $\delta$ for $A_1 =0.1$ and (**a**) $k_1 =0.1$; (**b**) $k_1 =0.4$; (**c**) $k_1 =0.9$. The values of $\delta$ are obtained by varying the wavenumber $k_2$ from 0 to 2 and the corresponding fixed $k_1$.

It may be interesting to display the existence area for various types of vector solitons on a plane in which one of the coordinates is the “walk-off” parameter $\delta$. In Fig. 5, these existence areas are shown on the $\kappa - \delta$ plane, for specific indicative values of the carrier wavenumbers $k_1$ and $k_2$. The color code for vector solitons is the same as that used in Figs. 3 and 4. The walk-off parameter is calculated by setting $k_1$ to a fixed value, and then varying $k_2$ from 0 to 2. In Fig. 5a, dark-bright (DB, blue color) vector solitons are dominant in the whole $\kappa$ interval. Small areas in which dark-dark (DD, green color) and bright-dark (BD, red color) however exist are visible for small positive values of $\delta$. Much richer patterns appear in Fig. 5b and c, for larger wavenumber $k_1$, in which all four types of vector solitons may exist in substantial parts of the $\kappa - \delta$ plane. In both figures, bright-bright (BB, black color) vector solitons exist in small areas of the plane at low $\kappa$ ($\kappa < 2.5$).

## Illustrative examples of vector solitons and their parameters

**Figure 6:**
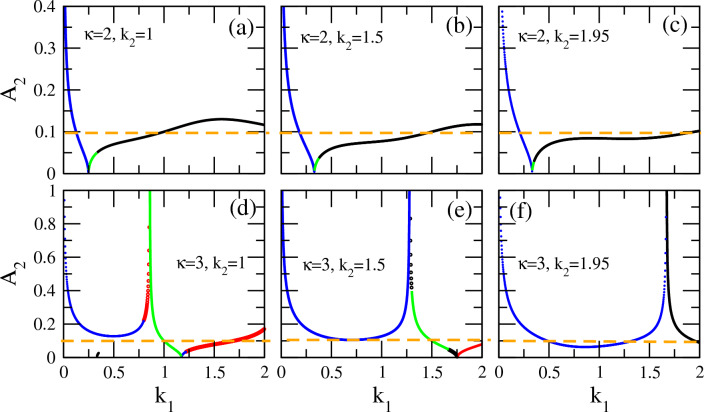
The amplitudes of the vector solitons components $A_1$ and $A_2$ as a function of the wavenumber $k_1$, calculated from the first of Eq. (21). The fixed amplitude $A_1 =0.1$ is indicated by the horizontal orange dashed line. (**a**) $\kappa =2$, $k_2 =1$; (**b**) $\kappa =2$, $k_2 =1.5$; (**c**) $\kappa =2$, $k_2 =1.95$; (**d**) $\kappa =3$, $k_2 =1$; (**e**) $\kappa =3$, $k_2 =1.5$; (**f**) $\kappa =3$, $k_2 =1.95$. Note that the same color code as in Figs. 3 and 4 has been adopted in all curves shown, i.e. black/red/blue/green color represents values prescribing BB/BD/DB/DD vector solitons, respectively.

It is tempting to investigate how the vector soliton characteristics vary when one of the wavenumbers of the carrier waves. e.g., $k_1$ varies (while the rest of the parameters as well as the amplitude $A_1$ remain fixed). In Fig. 6, the amplitude $A_2$ is plotted as a function of $k_1$ for two values of the $\kappa$, i.e., for $\kappa =2$ (upper panels) and $\kappa =3$ (lower panels), and for three different values of the wavenumber $k_2$ ($k_2 =1$, 1.5, 1.95). For the upper panels, the three values of $k_2$ correspond to three “cuts” (sections) at $k_2 =1$, 1.5, 1.95 of the $k_1 - k_2$ plane in Fig. 4. As obvious in this figure, for all three values, DB type vector solitons occur for low $k_1$, while upon increasing $k_1$ they successively turn to DD and then BB soliton pairs.

Figure 6a–c depicts the variation of the magnitude of (the amplitude) $A_2$ as well as its behavior when boundaries of areas of existence of different types of vector solitons are crossed. The three plots exhibit similar behavior: the amplitude $A_2$ takes very high values for low $k_1$ (DB vector solitons) which gradually decreases with higher $k_1$ and almost vanishes at the boundary between DB (blue) and DD (green) vector solitons (existence regions). As soon as this boundary is crossed, the amplitude $A_2$ increases again with increasing $k_1$, it reaches a maximum slightly above $A_1 =0.1$ and then decreases again slightly. Moreover, the second boundary crossing, i.e. between DD and BB vector solitons, is a smooth and continuous process as evident from the continuity of the green into the black part(s) of the plotted curve. interestingly, for $k_1 > 0.5$, the two amplitudes $A_1$ and $A_2$ are of the same order of magnitude.

The corresponding curves in the lower panels, obtained for $\kappa =3$, also exhibit a vanishing amplitude at the boundary between the areas of existence of DD to BD vector solitons. However, a different type of behavior is now witnessed, as the amplitude $A_2$ diverges at the boundary separating BD- from DD-type vector soliton regions (areas of existence): see Fig. 6d. In the former case, the variation of the amplitude $A_2$ through the corresponding boundary is smooth, while in the latter it is not. In the same figure, one also witnesses a smooth variation of $A_2$ through the boundary between DB (blue) and BD (red) vector solitons, as well as a divergence of the amplitude when crossing the boundary between the areas of existence of BD (red) and DD (green) vector solitons. Similar remarks can be made for Fig. 6e and f. In the latter figure, only one boundary crossing is visible for the $k_1$ interval shown.

**Figure 7:**
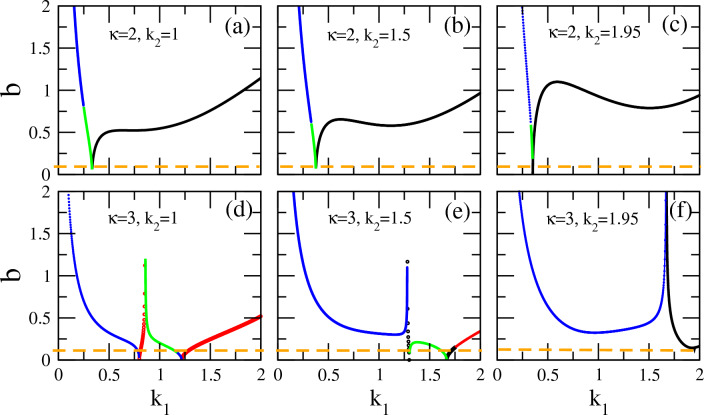
The width *b* of the vector soliton components as a function of the wavenumber $k_1$, calculated from the second of Eq. (21). The fixed amplitude $A_1 =0.1$ is indicated by the horizontal orange dashed line. (**a**) $\kappa =2$, $k_2 =1$; (**b**) $\kappa =2$, $k_2 =1.5$; (**c**) $\kappa =2$, $k_2 =1.95$; (**d**) $\kappa =3$, $k_2 =1$; (**e**) $\kappa =3$, $k_2 =1.5$; (**f**) $\kappa =3$, $k_2 =1.95$. Note that the same color code as in Figs. 3 and 4 has been adopted in all curves shown, i.e. black/red/blue/green color represents values prescribing BB/BD/DB/DD vector solitons, respectively.

The (common) width *b* of the vector soliton components associated to the amplitudes $A_2$ (Fig. 6) are presented in Fig. 7. The width *b* in all the sub-panels in Fig. 7 takes very high values at low $k_1$, up to $k_1 \simeq 0.1$, suggesting a very extended (spatially)—i.e. little localized—solution, but with high amplitude $A_2$. (Recall that $A_1$ is fixed in this figure.)

The behavior of *b* with $k_1$ increasing beyond 0.1 is then diversified for the two values of the spectral index shown: $\kappa =2$ (upper panels) and $\kappa =3$ (lower panels). Consider the former case first, i.e., that with $\kappa =2$. For $k_1$ increasing above 0.1, the width *b* decreases almost linearly but abruptly, passing smoothly through a boundary from DB (blue) to DD (green) vector soliton existence areas. At approximately $k_1 \simeq 0.3$, the width *b* reaches a very low value of the order of 0.05, while another boundary crossing between DD (green) to BB (black) vector solitons takes place at that point. For further increasing $k_1$, the width *b* increases again, its exact behavior however this time depends on the selected value of the second wavenumber $k_2$. In Fig. 7a, for $k_2 =1$, the width *b* after a linear increase it saturates to a value that is approximately constant around $b \simeq 0.5$. Then, for $k_1$ increasing above  0.8, the width *b* increases parabolically. For the two other values of $k_2$, i.e., for $k_2 =1.5$ and $k_2 =1.95$ shown in Fig. 7b and c, respectively, the width *b* as a function of $k_1$ does not form a plateau but instead it reaches a maximum, then a shallow minimum, and finally it start increasing parabolically upon further increasing $k_1$. Both the maximum and minimum values of *b* are higher for $k_2 =1.95$, as compared to the case with $k_2 =1.5$.

**Figure 8:**
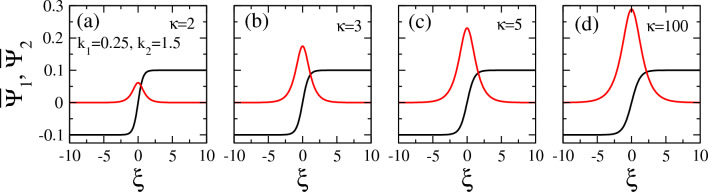
Variation of the vector soliton components as the spectral index $\kappa$ takes the values (**a**) $\kappa =2$; (**b**) $\kappa =3$; (**c**) $\kappa =5$; (**d**) $\kappa =100$, for a fixed point on the $k_1 - k_2$ plane, i.e., for $k_1 =0.25$ and $k_2 =1.5$. The envelops $\bar{\Psi }_1$ and $\bar{\Psi }_2$ are plotted as a function of $\xi$.

For $\kappa =3$, as shown in Fig. 7d–f, the width *b* of the vector solitons also decreases when $k_1$ increases further above 0.1 but not as abruptly as in the corresponding case with $\kappa =2$. In the $k_1$ interval shown in Fig. 7d–f, we observe 3, 3, and 1 boundary crossings, respectively (for $k_2 =1$, 1.5, and 1.95). Specifically, for $k_2 =1$ (Fig. 7d), the first boundary crossing appears at about $k_1 \simeq 0.8$ where from DB (blue) vector solitons one transits into BD (red) ones smoothly (i.e. the curve representing the width *b* is continuous) at very low values. The second crossing, from BD (red) to DD (green) type vector solitons occurs very close to the first one and is not smooth in this case, in the sense that *b* diverges at the boundary. A third crossing occurs at $k_1 \simeq 1.25$ where again the transition of *b* is smooth with very low values of *b* at the crossing point. The latter crossing is from DD (green) to BD (red) vector solitons. For $k_2 =1.5$ (Fig. 7e), a crossing between existence areas from DB (blue) to DD (green) vector solitons occurs at approximatelly $k_1 \simeq 1.25$ which is not smooth in *b*, since it diverges there. A second, smooth crossing occurs at around $k_1 \simeq 1.7$ in which *b* has low values and separates existence areas of DD (green) and BB (black) vector solitons. For that value of $k_2$, BB (black) vector solitons exist only for a very short interval of $k_1$, and the third crossing in this figure occurs at approximately $k_1 \simeq 1.75$ between existence areas of BB (black) to BD (red) vector solitons. This crossing is smooth and continuous in the width *b*. For $k_2 =1.95$ (Fig. 7f), a single crossing occurs between existence areas of DB (blue) and BB (black) vector solitons. The behavior of *b* as a function of $k_1$ has the same features as that of the amplitude $A_2$ shown in Fig. 6.

The divergence of the amplitude $A_2$ and the width *b* for low $k_1$ in Figs. 6 and 7 is due to the corresponding divergence of the coefficient $Q_{11}$, which appears in the numerator of both $\alpha$ and $\beta$ in Eq. (15). However, there is also a divergence at relatively large $k_1$ for $\kappa =3$, signaling the transition from one type of vector soliton to another. Specifically, in Figs. 6d and 7d a divergence in $A_2$ and *b* sets the boundary between a bright-dark (BD) and a dark-dark (DD) vector soliton at $k_1 =0.86$. That divergence occurs because both $\alpha$ and $\beta$ change sign through their denominator crossing the zero line. When this happens, a transition of a BD vector soliton (which exists for $\alpha >0$ and $\beta < 0$) to a DD vector soliton (which exists for $\alpha < 0$ and $\beta > 0$) takes place. (Note that the denominators of $\alpha$ and $\beta$ differ by an overall sign only.) Very close to the transition point, where the amplitude of the second component may be much larger than unity, the CNLS equations (8) and (9) are not expected to provide a valid description of vector solitons in our plasma fluid model.

### Extremely asymmetric waves emerging as vector soliton components

Note that, with reference to Figs. 6 and 7, there may exist boundaries between existence areas of different type of vector solitons in which the amplitude $\Psi _2$ of the envelope soliton component acquires large values, while its width *b* acquires very low values simultaneously. Such a case can be observed in Fig. 7e, for a value of $k_1$ slightly above 1.25 (green curves in Figs. 6e and 7e), for which DD vector solitons exist. For that $k_1 \simeq 1.3$, the width *b* acquires very low values while at the same time the amplitude $A_2$ of the second vector soliton component acquires values around $A_2 \simeq 0.4$, which are four (4) times larger that the corresponding amplitude $A_1 =0.1$ (fixed) of the first vector soliton component. Such a highly localized and high-amplitude envelope soliton can be characterized as “extremely asymmetric wave”, i.e. a large amplitude breather-like structure which co-exists (co-propagates) with an ordinary sister envelope soliton (pulse). Of course, the specific choice of values makes the components of the (DD in the described case) vector soliton highly a-symmetric. This possibility, as well as the stability and geometry of extremely asymmetric vector soliton (pairs) will be analyzed in detail in a future a work.

**Figure 9:**
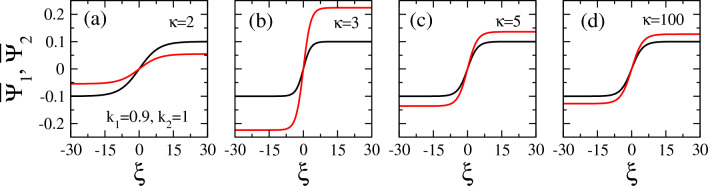
Same as Fig. 8 but for $k_1 =0.9$ and $k_2 =1$.

**Figure 10:**
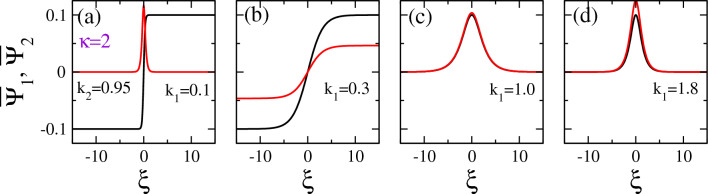
Vector solitons along a “cut” of the $k_1 - k_2$ plane in Fig. 3a at $k_2 =0.95$ for $\kappa =2$ and (**a**) $k_1 =0.1$; (**b**) $k_1 =0.3$; (**c**) $k_1 =1$; (d) $k_1 =1.8$. We observe a DB vector soliton in (**a**), a DD vector soliton in (**b**), and a BB vector solitons in (**c**,**d**).

**Figure 11:**
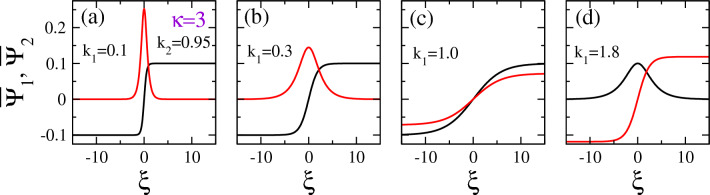
Vector solitons along a “cut” of the $k_1 - k_2$ plane in Fig. 3b at $k_2 =0.95$ for $\kappa =3$ and (**a**) $k_1 =0.1$; (**b**) $k_1 =0.3$; (**c**) $k_1 =1$; (**d**) $k_1 =1.8$. We observe a DB vector soliton in (a)-(b), a DD vector soliton in (**c**), and a BD vector soliton in (**d**).

In Figs. 8, 9, 10, 11, several illustrative examples of all the four types of vector solitons which may exist in certain areas on the $k_1 - k_2$ plane, are provided for several values of the spectral index $\kappa$ and the wavenumbers $k_1$ and $k_2$. The first two of these figures, in particular, i.e., Figs. 8 and  9, provide illustrative examples of DB and DD vector solitons, respectively, for $\kappa =2$, 3, 5, and 100. E.g., in Fig. 8, the values for the two wavenumbers were chosen to be $k_1 =0.25$ and $k_2 =1.5$ (for all subfigures), while $\kappa$ varies as shown on the figure. In this particular case, the values of $k_1$ and $k_2$ favor the existence of DB vector solitons for any $\kappa >2$. Note that the amplitude of the envelop soliton component (black curve), $\Psi _1$ , $A_1$, is fixed to 0.1, and thus it is the same in Fig. 8a through d, even though its width *b* does change slightly its value. The second vector soliton component (red curve), $\Psi _2$, has a varying amplitude which increases considerably with increasing $\kappa$, as can be observed in Fig. 8 (e.g., from $A_2 \simeq 0.06$ in Fig. 8a to $A_2 \simeq 0.29$ in Fig. 8d.

Similarly, in Fig. 9, the same plots of vector solitons as those presented before in Fig. 8 are shown but for a different $k_1$ and $k_2$ pair of values on the $k_1 - k_2$ plane, i.e., $k_1 = 0.9$ and $k_2 =1$. for this choice of the $k_1$ and $k_2$ pair, we get DD vector solitons for all the values of the spectral index $\kappa$ considered. Of course the width of the envelopes *b* and the amplitude of the vector soliton component $\Psi _2$, $A_2$, change considerably with $\kappa$. Interestingly, the width *b* of both soliton components is rather large for an extreme (strongly non-Mawxellian) value $\kappa =2$ (Fig. 9a). Also, the amplitude $A_2$ is very low in this case, i.e., $A_2 =0.05$. In Fig. 9b, however, for $\kappa =3$, the width *b* decreases considerably, so that the soliton components become narrower and thus highly localized, while at the same time the amplitude $A_2$ increases to more than 0.22. With further increasing $\kappa$, both *b* and $A_2$ gradually and slowly decrease, while they tend to become equal to each other for very large values of $\kappa$, e.g., for $\kappa =100$ in Fig. 9d.

The vector solitons shown in Figs. 10 and 11 are obtained for spectral index $\kappa =2$ and 3, respectively, and different pairs of wavenumbers $k_1$ and $k_2$, along $k_2 =0.95$ on the $k_1 - k_2$ plane. In Fig. 10a, a DB vector soliton is shown for $k_1 =0.1$ and $k_2 =0.95$, which are both very narrow (small width *b*) and have roughly the same amplitude, i.e., $A_1 \simeq A_2 \simeq 0.1$. A DD vector soliton is shown in Fig. 10b for $k_1 =0.3$ and $k_2 =0.95$, where the amplitude $A_1$ is twice the amplitude $A_2$. In Fig. 10c and d, two BB vector solitons are shown for $k_1 =1.0, k_2 =0.95$, and $k_1 =1.8, k_2 =0.95$, respectively. The two components of these vector solitons do not differ very much and, in one case (Fig. 10d), the two amplitudes $A_1$ and $A_2$ as well as their width *b* are practically the same. This seems reasonable since the values of $k_1$ and $k_2$ in this case are very close together and thus close to the curve $k_1 =k_2$ on the $k_1 - k_2$ plane, where the CNLS system becomes nearly symmetric.

In Fig. 11, several vector solitons are shown for $\kappa =3$ and the same pairs of values of the wavenumbers $k_1$ and $k_2$ as those used in Fig. 10. Here, a narrow (small *b*) highly localized DB vector soliton is shown in Fig. 11a, whose $\Psi _2$ component has a rather high amplitude $A_2 \simeq 0.3$, about three times the amplitude $A_1$. In Fig. 11b we have another DB vector solitons whose components are however much wider than the previous one (large *b*) and $A_2$ is roughly $1.5 A_1$. In Fig. 11c, we have a DD vector soliton whose components have similar amplitudes but they are rather wide (large *b*), Next, in Fig. 11d, we have two BD vector solitons, whose components exhibit comparable amplitude.

**Figure 12:**
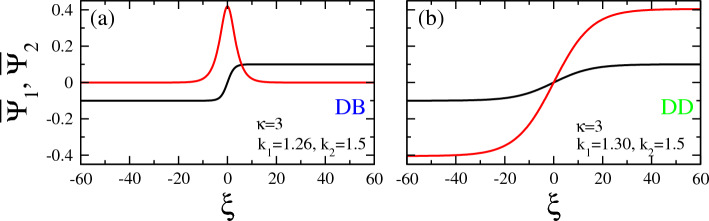
Extreme amplitude (asymmetric) vector solitons obtained around the boundary of the area of existence of dark-bright (DB, blue color) and dark-dark (DD, green color) in Fig. 3b for spectral index $\kappa =3$, $k_2 =1.5$, and (**a**) $k_1 =1.26$; (**b**) $k_1 =1.30$. In both plots, the amplitude of the second component of the vector soliton (bright and dark, respectively) is significantly larger than that of the first one.

An illustrative example of vector solitons with an extreme amplitude component is shown in Fig. 12. In that figure, two such vector solitons obtained for the same spectral index $\kappa =3$ and wavenumber of the second carrier wave $k_2 =1.5$ are shown for slightly different values of $k_1$. These parameter values correspond to points very close to the boundary of the area of existence of dark-bright (DB, blue color) and dark-dark (DD, green color) of Fig. 3b for $\kappa =3$. At these points, the second components of the existing vector solitons are expected from Fig. 6e to have a high amplitude as compared with the first one. In Fig. 12a, for $k_1 =1.26$, in which the vector soliton is of the DB type, the amplitude of the second (bright) component which is shown in red color is about four times larger than that of the first one. The same remark holds for the amplitude of the second (dark) component shown in red color in Fig. 12b, for $k_1 =1.30$, is again about four times larger than the first one. Note the both in Fig. 12a and b the first component of the corresponding vector soliton is shown in black color and it is of the dark type. Thus, in this particular case one may switch from a certain type of vector soliton with a large component to another type (i.e., bright to dark, in this case) by a small shift in one of the wavenumbers, thus crossing a boundary between adjacent areas in the existence diagram. Note that the extreme amplitude component is the one that changes type in this case, i.e. from bright in Fig. 12a to dark in Fig. 12b. (Recall that the amplitude of the first component in all vector solitons presented here is numerically fixed to $A_1 =0.1$, for comparison and reference).

**Figure 13:**
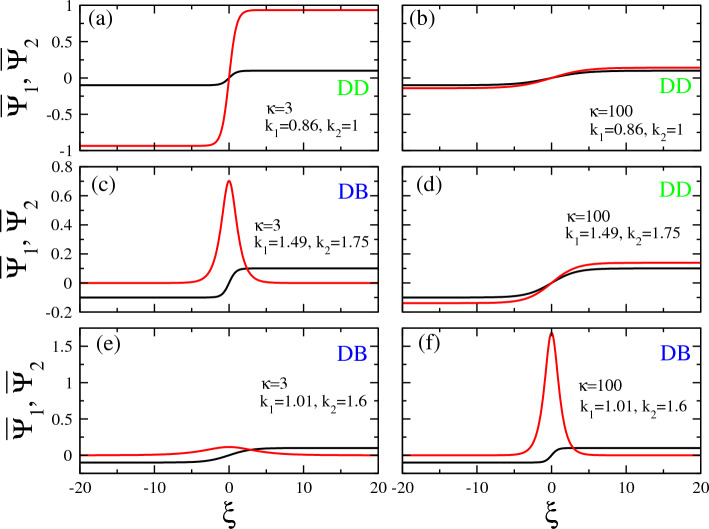
Comparison among representative vector soliton pairs obtained for small and large values of the spectral index $\kappa$, i.e., for $\kappa =3$ (left panels; strongly non-thermal distribution): (**a**) $k_1 =0.86$, $k_2 =1$; (**c**) $k_1 =1,49$, $k_2 =1.75$; (**e**) $k_1 =1.01$, $k_2 =1.6$, and $\kappa =100$ (right panels; quasi-Maxwellian): (**b**) $k_1 =0.86$, $k_2 =$; (**d**) $k_1 =1.49$, $k_2 =1.75$; (**f**) $k_1 =1.01$, $k_2 =1.6$. The vertical scales are the same for all panels in each row, to facilitate comparison. The horizontal scales are the same for all panels.

Further examples of vector solitons with an extreme amplitude component are shown in Fig. 13, in which the effect of extreme variation of the spectral index $\kappa$ is illustrated. The two panels in each row are obtained for the same pairs of the wavenumbers $k_1$ and $k_2$ but for different $\kappa$. In all left panels, the value of spectral index $\kappa =3$ has been used, while in all right panels a large value of $\kappa$ is used ($\kappa =100$) for which the electron distribution practically coincides with a Maxwell-Boltzmann one. In Fig. 13b, for $\kappa =100$, the selected values of $k_1$ and $k_2$ are such that the vector soliton is of the dark-dark (DD) type whose components have almost the same (relatively low) amplitude. However, as the spectral index is decreased to the relatively small value $\kappa =3$ in Fig. 13a, the amplitude of the second component increases significantly with respect to that of the first one, although the the vector soliton type (DD) remains the same. Thus, by decreasing $\kappa$, the resulting increase in the suprathermal electron population provides the necessary energy for the emergence of a dark type extreme amplitude excitation shown in red color.

In the second row, i.e. in Fig. 13c and d, we observe the emergence of an extreme amplitude *bright* type component upon decreasing the value of $\kappa$, respectively. It appears that moving far from Maxwellian equilibrium results in energizing the electrons, that supply the energy required to excite a large amplitude “breather” type envelope structure seven (!) times higher than the sister component. A different trend is witnessed in Fig. 13e and f. In this case, somehow counter-intuitively, a small-amplitude dark (wave 1)/large amplitude bright (wave 2) vector soliton excited for $\kappa =100$—see 13f—is in fact suppressed in amplitude, upon reducing the spectral index to $\kappa =3$—see Fig. 13e—however without changing structural type (DB).

**Figure 14:**
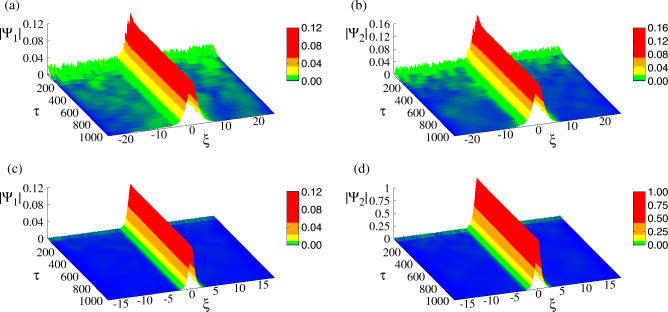
Temporal evolution of bright-bright vector solitons profiles obtained from numerical simulations using Eqs. (8) and (9) with initial condition from Eq. (13) to which small random noise is added. The profiles in (**a**) and (**b**) (resp. (**c**) and (**d**)) for $\bar{\Psi }_1$ and $\bar{\Psi }_2$ are obtained for $k_1 =1.5$ and $k_2 =1$ (resp. $k_1 =1.673$ and $k_2 =1.95$). The parameters of the vector soliton in (**a**) and (**b**) (resp. in (**c**) and (**d**)) are $A_1 =0.1$, $A_2 \simeq 0.129$, $b \simeq 0.759$ (resp. $A_1 =0.1$, $A_2 \simeq 0.964$, $b \simeq 2.13$).

In order to address the stability of the obtained vector solitons, linear stability analysis corroborated by direct numerical simulations and/or (numerical) spectral stability analysis should be used to obtain the parameter regimes which can support stable vector solitons. Further analytical approaches may involve methods applied, e.g., to birefringent optical fibers^62^ or the application of the Vakitov-Kolokolov criterion^63^. The stability analysis of the various types vector solitons in plasmas is certainly important and worth studying on its own right and it is a matter of future work. Here, stability is demonstrated using direct numerical simulations for two bright-bright (BB) vector solitons; the parameters are chosen so that the components of one of them have similar amplitude while the components of the other are highly asymmetric (e.g. the amplitude of the second component is much higher than that of the first).

These two cases are illustrated in Fig. 14 in which (a) and (b) show the temporal evolution of the $|\bar{\Psi }_1|$ and $|\bar{\Psi }_2|$ profiles for the first set of parameters, while (c) and (d) those for the second set of parameters (see caption in Fig. 14). These simulations are initialized with the corresponding analytical solutions given in Eq. (13), to which small random noise was incorporated. In Fig. 14a and b that random noise is relatively large so that it is clearly visible for small $\tau$. At both ends of of the $\xi$ interval, dissipation has been added by hand to remove the excess energy introduced by the noise. In this way, the excess energy leaves the system and at large $\tau$ the profiles practically coincide with the analytical ones. We have also checked that at large $\tau$ the norms of the two components $|\bar{\Psi }_1|^2$ and $|\bar{\Psi }_2|^2$ saturate to a constant value.

## Discussion and conclusions

We have considered the simultaneous propagation of a pair of (nonlinearly interacting) electrostatic wavepackets in a collisionless unmagnetized electron-ion plasma, from first principles. The wavepackets are not identical, in the sense that both their amplitudes and (carrier) wavenumbers $k_1$ and $k_2$ are allowed to differ. Adopting a Newell type multiple (time and space) scales technique, a pair of CNLS equations was derived. A standard non-magnetized plasma fluid model was adopted, for simplicity, comprising cold inertial ions evolving against an inertialess electron background. The electron population was assumed to obey a kappa-type distribution, which is characterized by the spectral index parameter $\kappa$, a situation often occurring in space plasmas (with typical values of $\kappa$ usually ranging from 2 to 6). As the kappa distribution diverges significantly from a Maxwell-Boltzmann distribution, the highly energetic (suprathermal) electron component results in significant modification of the modulated wavepackets characteristics and interactions thereof. We have investigated the modulational (in)stability profile of the coupled wavepacket pair, focusing on its dependence on the electron spectral index ($\kappa$). We have shown that various types of vector solitons may exist in different areas on the $k_1 - k_2$ plane, while their shape depends on (the value of) $\kappa$. The strongest variation is observed in the interval of $\kappa$ from 2 to 3.

The six coefficients of the CNLS equations, i.e., the dispersion coefficients $P_j$, the nonlinearity coefficients $Q_{jj}$, and the nonlinear coupling coefficients $Q_{ij}$ (with $i\ne j$), are given by complicated algebraic expressions (see Supplementary Information) as functions of the wavenumbers $k_1$ and $k_2$ and the spectral index $\kappa$. For arbitrary values of $k_1$ and $k_2$ ($\ne k_1$), these coefficients do not possess any particular symmetry, hence the generalized system of CNLS equations thus obtained is most likely non-integrable, in the general case. (Obviously, the integrable Manakov case is recovered if $k_1 = k_2$.) The asymmetry of the CNLS equations does not prevent one from obtaining vector soliton solutions, as combinations of bright (B) and dark (D) envelopes structures, i.e. of BB, BD, DB or DD type, each of which will occur in particular areas on the $k_1 - k_2$ parameter plane.

The “area of existence” of these vector solitons exhibits strong variation with respect to $\kappa$, in particular for values of $\kappa$ between 2 and 3. For $\kappa$ close to 2, BB vector solitons exist in the largest part of the plane, while BD and DB vector solitons exist only for low $k_1$ and $k_2$, respectively. Also, DD vector solitons exist in narrow areas between BB-BD and BB-DB vector solitons. However, for increasing $\kappa$, the area of existence of BD and DB vector solitons increases at the expense of the area of BB vector solitons. At the same time, the area of existence of DD vector solitons also increases. For $\kappa$ greater than 3, the pattern of the areas of existence of the four vector solitons change only slightly with $\kappa$.

The vector soliton parameters, i.e., their amplitude and width, both of which have been calculated analytically, also vary significantly with varying $k_1$, $k_2$ and $\kappa$. It is interesting to see how the transition between different types of vector solitons occurs, upon varying one of these parameters, keeping the remaining two parameters fixed. As illustrated in the figures above, this transition between different types of vector solitons can be either smooth, or associated with a divergence of, say, the amplitude of one wave at the transition point, i.e. at the boundary separating areas of existence of different types of vector solitons.

Of particular interest is the situation in which, close to a transition point where a soliton parameter diverges, the amplitude of one of the components may acquire extreme values, i.e. far exceeding its sister wave’s amplitude, thus forming what could be characterized as an extreme amplitude wave (component) pair. These highly asymmetric vector solitons are a peculiarity which is attributed to the general asymmetry of the CNLS equations. The investigation of the stability of these vector solitons using semi-analytic and numerical methods will be a subject of future work.

Focusing on the role of suprathermal electrons, our investigation has shown that the spectral index $\kappa$ affects significantly the modulational (in)stability profile of the CNLS system as well as the characteristic of vector soliton types that may be sustained in the plasma. For smaller $\kappa$, i.e., as the electron distribution deviates from the Maxwellian one, the areas on the parameter planes in which modulational instability appears become larger, while at the same time the growth rate becomes higher in those areas (i.e., enhancing the instability). Concerning vector solitons, a variation in the value of $\kappa$ modifies the existence diagram in parameter space, in which different types of vector solitons may occur. The most prominent variation occurs below $\kappa =3$, where DB, BD, and DD vector solitons exist in substantial areas of the $k_1 - k_2$ plane, down to $\kappa =2$, where BB vector solitons become dominant.

The existence of all four types of vector solitons on a parameter plane involving the spectral index $\kappa$ and the walk-off parameter $\delta$ was also illustrated and discussed. Notably, for the particular plasma model considered here, the parameter $\delta$ takes low values (in fact, the lower $\kappa$, the smaller the range of $\delta$), which are thus not expected to affect the formation or the stability of vector solitons.

The formation of solitary waves/solitons is a phenomenon that commonly occurs in space plasmas, e.g., in the solar wind and in planetary magnetospheres; cf. observations of electrostatic solitary waves by the Cluster satellites^64,65^ (and references therein). As one example, electrostatic solitary waves have been observed in the Earth’s magnetopause^66,67^, and their theoretical interpretation requires resorting to multicomponent plasma fluid models^68^. Remarkably, envelope structures (breathers, rogue waves) modeled by the NLS equation have been realized in laboratory plasmas, little more than a decade ago^69^.

Based on earlier considerations, where modulational instability has been proposed as an intermediate stage between amplitude modulation of a Stokes wave and higher-order effects leading to rogue wave formation^70^, we anticipate that the creation of extreme amplitude soliton-pair structures predicted by our model may provide an effective framework as a precursor towards freak wave occurrence in relation with electtostatic plasma modes.

Our work aims at providing a platform for modeling solitons/solitary waves in space plasmas, where modulated envelope pairs may emerge from two or more interacting nonlinear waves. In a wider context, our results will be valuable in other disciplines where wavepackets may propagate in nonlinear dispersive media, including—but not being limited to—hydrodynamics, nonlinear (fiber) optics and telecommunications (signal transmission via optical pulses), to mention a few.

### Supplementary Information

Supplementary Information.

## Data Availability

The datasets generated during and/or analysed during the current study are available from the corresponding author upon reasonable request.
