# Author Correction: Electrostatic wave interaction via asymmetric vector solitons as precursor to rogue wave formation in non-Maxwellian plasmas

**DOI:** 10.1038/s41598-024-58477-x

**Published:** 2024-04-04

**Authors:** N. Lazarides, Giorgos P. Veldes, D. J. Frantzeskakis, Ioannis Kourakis

**Affiliations:** 1 Department of Mathematics, Khalifa University of Science and Technology, P.O. Box 127788, Abu Dhabi, United Arab Emirates; 2 Department of Physics, University of Thessaly, 35100 Lamia, Greece; 3 Department of Physics, National and Kapodistrian University of Athens, Zografou, 15784 Athens, Greece; 4 Space & Planetary Science Center, Khalifa University of Science and Technology, P. O. Box 127788, Abu Dhabi, United Arab Emirates; 5 Hellenic Space Center, Leoforos Kifissias 178, Chalandri, 15231 Athens, Greece

Correction to: *Scientific Reports* 10.1038/s41598-024-52431-7, published online 25 January 2024

The original version of this Article contained an error in the analytical expression in the section Modulational instability: compatibility condition and growth rate, where “K^4^” was omitted.

$$\Omega _c^4 =4 P_1 P_2 Q_{12} Q_{21} \Psi _{1,0}^2 \Psi _{2,0}^2$$

now reads:

$$\Omega _c^4 =4 P_1 P_2 Q_{12} Q_{21} \Psi _{1,0}^2 \Psi _{2,0}^2 K^4$$

The original Article has been corrected.

