# Supplementary material for: Electrostatic wave interaction via asymmetric vector solitons as precursor to rogue wave formation in non-Maxwellian plasmas
Source: Sci Rep. 2024 Jan 25;14:2150. doi: 10.1038/s41598-024-52431-7 (PMC10810890; doi:10.1038/s41598-024-52431-7)
Supplement: Supplementary file 1 — Supplementary Information. [file 41598_2024_52431_MOESM1_ESM.pdf]

Supplementary Information for "Electrostatic wave interaction via asymmetric vector solitons as precursor to rogue wave formation in non-Maxwellian plasmas"

N. Lazarides[1], Giorgos P. Veldes[2], D.J. Frantzeskakis[3] and Ioannis Kourakis[1,4,3,5]

[1]Department of Mathematics, Khalifa University of Science and Technology, P.O. Box 127788, Abu Dhabi, United Arab Emirates
[2] Department of Physics, University of Thessaly, Lamia 35100, Greece
[3] Department of Physics, National and Kapodistrian University of Athens, GR-15784 Zografou, Athens, Greece
[4] Space & Planetary Science Center, Khalifa University of Science and Technology, P. O. Box 127788, Abu Dhabi, United Arab Emirates
[5] Hellenic Space Center, Leoforos Kifissias 178, Chalandri, GR-15231 Athens, Greece

Nonlinearity Coefficients in the CNLS Eqs. (1) and (2) in the Main Text – Analytical Expressions

Note that each of the indices i and j takes either of the values 1 and 2, wherever it appears in the following.

The (four) nonlinearity coefficients appearing in the CNLS Eqs. (1) and (2) in the main text are given by the expressions:

$$Q_{ij} = \frac{\omega_j}{2k_j^2}\tilde{Q}_{ij}, \tag{S1}$$

where ω_j is the angular frequency (of the $j-$th carrier wave): note that this is essentially a function of the wavenumber k_j, given by the dispersion relation Eq. (4) in the main text. The quantities $\tilde{Q}_{ij}$ appearing above are defined below.

The self-modulation coefficients (for $i = j =$ either 1 or 2) are given by:

$$\tilde{Q}_{jj} = -2\frac{k_j^3}{\omega_j}\left(C_{u,2,j}^{(2)} + C_{u,2,j}^{(0)}\right) - k_j^2\left(C_{n,2,j}^{(2)} + C_{n,2,j}^{(0)}\right) + 2c_2\omega_j^2\left(C_{\phi,2,j}^{(2)} + C_{\phi,2,j}^{(0)}\right) + 3c_3\omega_j^2, \tag{S2}$$

while the cross-coupling coefficients (for $i \neq j$) are given by:

$$\tilde{Q}_{12} = -2\frac{k_1^3}{\omega_1}C_{u,2,2}^{(0)} - k_1\frac{k_2}{\omega_2}\left(\omega_1\frac{k_2}{\omega_2} + k_1\right)\left(C_{u,2,+}^{(1)} + C_{u,2,-}^{(1)}\right) - k_1^2C_{n,2,2}^{(0)} - \omega_1 k_1\frac{k_2}{\omega_2}\left(C_{n,2,+}^{(1)} + C_{n,2,-}^{(1)}\right)$$
$$+2c_2\omega_1^2\left(C_{\phi,2,2}^{(0)} + C_{\phi,2,+}^{(1)} + C_{\phi,2,-}^{(1)}\right) + 6c_3\omega_1^2, \tag{S3}$$

$$\tilde{Q}_{21} = -2\frac{k_2^3}{\omega_2}C_{u,2,1}^{(0)} - k_2\frac{k_1}{\omega_1}\left(\omega_2\frac{k_1}{\omega_1} + k_2\right)\left(C_{u,2,+}^{(1)} + C_{u,2,-}^{(1)}\right) - k_2^2C_{n,2,1}^{(0)} - \omega_2 k_2\frac{k_1}{\omega_1}\left(C_{n,2,+}^{(1)} + C_{n,2,-}^{(1)}\right)$$
$$+2c_2\omega_2^2\left(C_{\phi,2,1}^{(0)} + C_{\phi,2,+}^{(1)} + C_{\phi,2,-}^{(1)}\right) + 6c_3\omega_2^2. \tag{S4}$$

The various coefficients entering the latter expressions are given by:

$$C^{(2)}_{n,2,j} = \frac{(k_j^2+c_1)}{6k_j^2}\left[3(4k_j^2+c_1)(k_j^2+c_1)-2c_2\right], \tag{S5}$$

$$C^{(2)}_{u,2,j} = \frac{\sqrt{k_j^2+c_1}}{6k_j^2}\left[3(k_j^2+c_1)(2k_j^2+c_1)-2c_2\right], \tag{S6}$$

$$C^{(2)}_{\phi,2,j} = \frac{1}{6k_j^2}\left[3(k_j^2+c_1)^2-2c_2\right], \tag{S7}$$

$$\begin{aligned}
C^{(1)}_{n,2,\pm} &= \frac{1}{D_\pm}\left\{\left[(k_1\pm k_2)^2+c_1\right]\left[(k_1\pm k_2)f_2^{(\pm)}+(\omega_1\pm\omega_2)f_1^{(\pm)}\right]+(k_1\pm k_2)^2 f_3^{(\pm)}\right\},\\
C^{(1)}_{u,2,\pm} &= \frac{1}{D_\pm}\left\{(k_1\pm k_2)\left[f_1^{(\pm)}+(\omega_1\pm\omega_2)f_3^{(\pm)}\right]+(\omega_1\pm\omega_2)\left[(k_1\pm k_2)^2+c_1\right]f_2^{(\pm)}\right\},\\
C^{(1)}_{\phi,2,\pm} &= \frac{1}{D_\pm}\left\{(\omega_1\pm\omega_2)\left[f_1^{(\pm)}+(\omega_1\pm\omega_2)f_3^{(\pm)}\right]+(k_1\pm k_2)f_2^{(\pm)}\right\},
\end{aligned} \tag{S8}$$

where

$$D_\pm = (k_1\pm k_2)^2-(\omega_1\pm\omega_2)^2\left[(k_1\pm k_2)^2+c_1\right]. \tag{S9}$$

and

$$f_1^{(+)} = -(k_1+k_2)\frac{k_1k_2}{\omega_1\omega_2}\left(\frac{k_1}{\omega_1}+\frac{k_2}{\omega_2}\right), \tag{S10}$$

$$f_2^{(+)} = -(k_1+k_2)\frac{k_1k_2}{\omega_1\omega_2}, \tag{S11}$$

$$f_3^{(+)} = +2c_2, \tag{S12}$$

$$f_1^{(-)} = -(k_1-k_2)\frac{k_1k_2}{\omega_1\omega_2}\left(\frac{k_1}{\omega_1}+\frac{k_2}{\omega_2}\right), \tag{S13}$$

$$f_2^{(-)} = -(k_1-k_2)\frac{k_1k_2}{\omega_1\omega_2}, \tag{S14}$$

$$f_3^{(-)} = +2c_2. \tag{S15}$$

$$C^{(0)}_{n,2,j} = \left(c_1C^{(0)}_{\phi,2,j}+2c_2\right), \qquad C^{(0)}_{u,2,j} = \frac{1}{v_{g,j}}\left[C^{(0)}_{\phi,2,j}+\frac{k_j^2}{\omega_j^2}\right], \qquad C^{(0)}_{\phi,2,j} = \rho_j\frac{1}{1-c_1v_{g,j}^2}, \tag{S16}$$

where

$$\rho_j = -(k_j^2+c_1)-2c_1+2c_2v_{g,j}^2. \tag{S17}$$

Finally, recall that the (electron distribution related) coefficients c_1, c_2 and c_3 were defined in the main text: see Eqs. (6)-(8).
